# Supplementary material for: A Bayesian brain model of adaptive behavior: an application to the Wisconsin Card Sorting Task
Source: PeerJ. 2020 Nov 30;8:e10316. doi: 10.7717/peerj.10316 (PMC7713598; doi:10.7717/peerj.10316)
Supplement: Supplemental Information 1 [file peerj-08-10316-s001.docx]

**A Bayesian brain model of adaptive behavior: An application to the Eisconsin Card Sorting Task**

**Supplementary Material**

**Marco D’Alessandro, Stefan T. Radev, Andreas Voss, and Luigi Lombardi**

Corresponding author: Marco D’Alessandro ([marco.dalessandro@unitn.it](mailto:marco.dalessandro@unitn.it))

In what follows, we provide the full joint posterior densities of the two cognitive parameters, namely, flexibility (**λ**) and information loss (**δ**), together with the mean coordinates (dotted lines) used as point estimates to recover information-theoretic quantities in the main text.


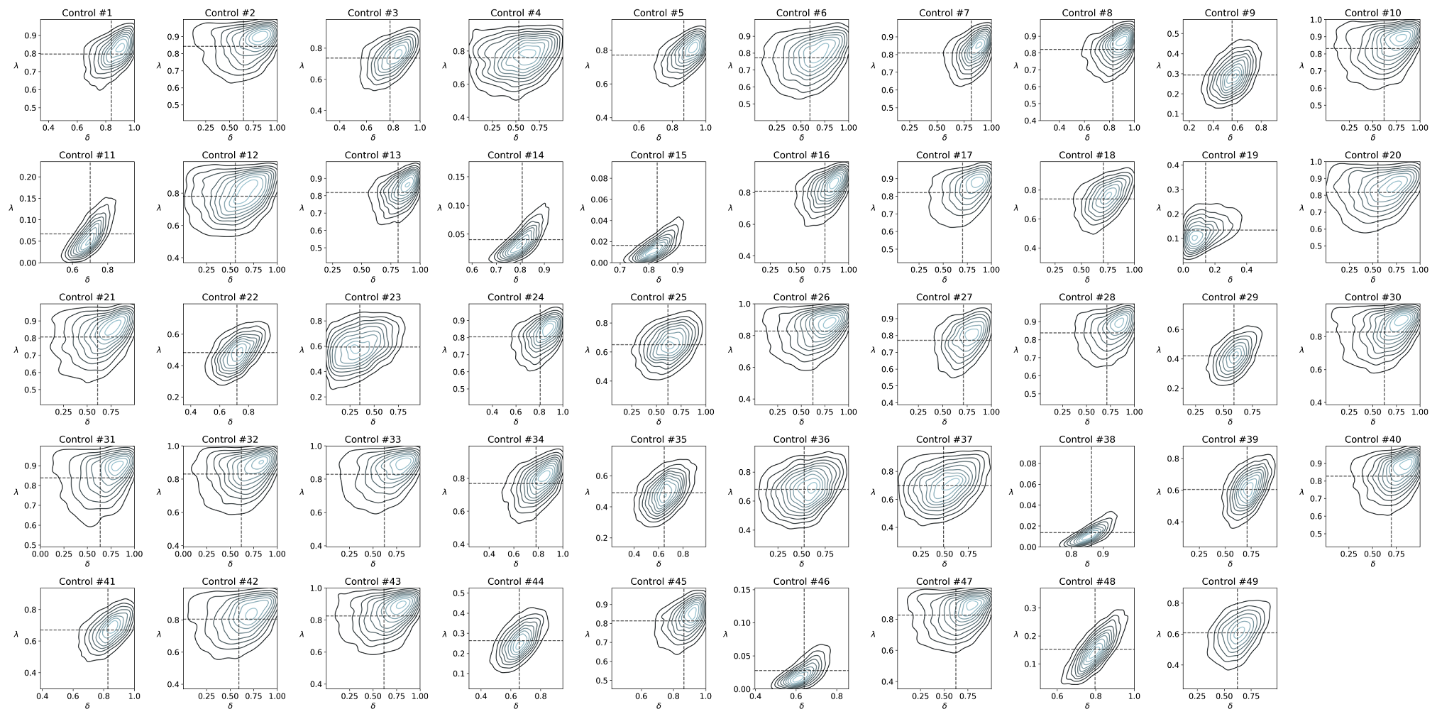


**Healthy individuals.** The figure shows the joint posterior density of the two cognitive parameters for each participant in the control group. Dotted lines show the distribution mean.


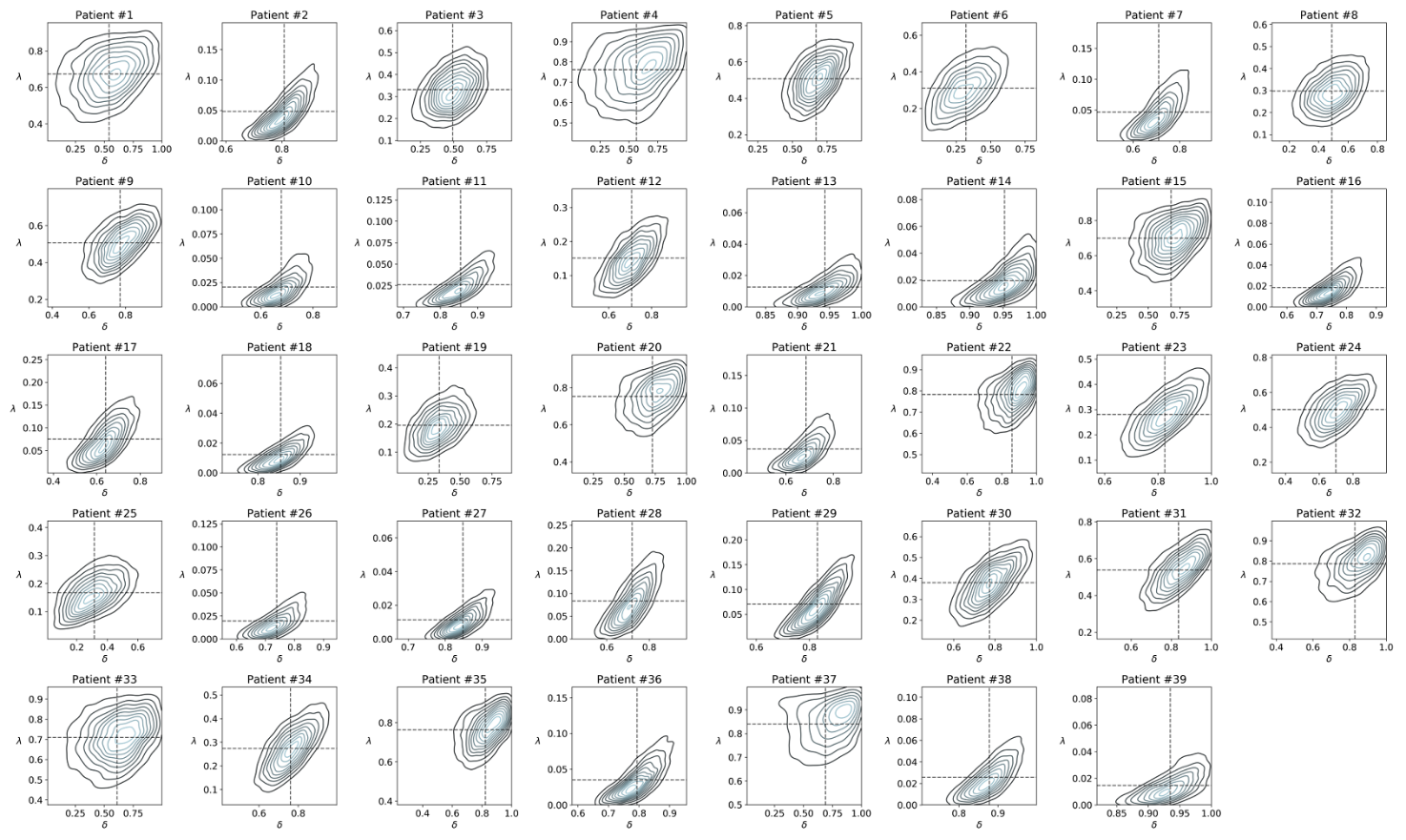


**Sudstance dependent individuals.** The figure shows the joint posterior density of the two cognitive parameters for each patient in the SDI group. Dotted lines show the distribution mean.
